# Supplementary material for: Through-container quantitative analysis of hand sanitizers using spatially offset Raman spectroscopy
Source: Commun Chem. 2021 Sep 2;4:126. doi: 10.1038/s42004-021-00563-6 (PMC9814617; doi:10.1038/s42004-021-00563-6)
Supplement: Supplementary file 1 — Description of Additional Supplementary Files [file 42004_2021_563_MOESM1_ESM.pdf]

Description of Additional Supplementary Files

File Name: Supplementary Data 1

Description: Contents, formulations, and container types of samples used in MCR and regression models. ST: Semi-transparent, T: Transparent, O: Opaque.
