# Supplementary material for: Through-container quantitative analysis of hand sanitizers using spatially offset Raman spectroscopy
Source: Commun Chem. 2021 Sep 2;4:126. doi: 10.1038/s42004-021-00563-6 (PMC9814617; doi:10.1038/s42004-021-00563-6)
Supplement: Supplementary file 2 — Supplementary Information [file 42004_2021_563_MOESM2_ESM.pdf]

Supplementary Information:

Through-Container Quantitative Analysis of Hand Sanitizers Using SORS and Support Vector Regression

Nirzari Gupta, Jason Rodriguez, Huzeyfe Yilmaz*

Division of Complex Drug analysis, U.S. Food and Drug Administration, St Louis, Missouri 63110, USA

*Corresponding author huzeyfe.yilmaz@fda.hhs.gov

Supplementary Methods

Statistical analysis and modeling. All spectra were baseline corrected using automatic Whittaker filter and normalized by area. We used multivariate curve resolution-alternating least squares (MCR-ALS) for visual demonstration of effectiveness of spatial offset. Our rationale for electing to use MCR-ALS over more commonly employed MVAs such as Principal Component Analysis (PCA) was based on the simplicity of the components. While PCA could be useful for exploration, MCR-ALS provided scientifically meaningful components (loadings) that could be interpreted by non-chemometricians. Furthermore, due to the construction of the MCR-ALS problem, it has found excellent use in analysis of chemical mixtures and pharmaceutical applications:[1, 2]

$$\mathbf{D} = \mathbf{C}\mathbf{S}^{\mathbf{T}} + \mathbf{E}$$

for the dataset $\mathbf{D}$ containing a mixture of pure components $\mathbf{S}^{\mathbf{T}}$ at concentrations $\mathbf{C}$. A non-negativity constraint is applied to each least square step to C and/or S^T on the constituent profile. This allows MCR components to be easily attributed to analyte spectra. Here, in-house hand sanitizer formulations with four different alcohols (ethanol, methanol, 2-propanol, and 1-propanol) in four different material types of containers (glass, PET, PE, and PP) with varying opacity were tested. MCR models with 7 components (with 98.8% cumulative fit) for zero spectra and 4 components (with 99.2% cumulative fit) were constructed for SORS. Number of components were based on the number of Raman-active container materials and types of alcohol involved in this study. We used venetian blinds for cross validation, with 10 numbers of data splits and 2 samples per blind for all models in this work.

Data acquisition (Agilent RapID).

1. Polynomial baseline correction: There are multiple built-in options in RapID. Polynomial baseline correction was selected as it provided a more realistic Raman spectra, was less aggressive and allowed observation of shoulders in alcohol mixtures.

2. Scaled subtraction: A built-in algorithm that subtracts the zero (container) spectrum from the offset (contents + container) spectrum in RapID was used. The resulting spectrum was representative of contents.

Algorithms and pre-processing (PLS toolbox).

1. Whittaker baseline correction: The Automatic Whittaker Filter method[3] was used to eliminate any remnant container artefacts in the scaled-subtracted spectra.

- Lambda - the parameter that controls the amount of curvature allowed for the baseline was selected as 100.
- P – the parameter that governs the extent of asymmetry required of the fit was selected as 0.001.

2. Normalization: Spectra were normalized (area = 1) to eliminate intensity fluctuations caused by container opacity.

3. MCR:

- ALS non-negativity: True least-squares solution (fasternnls – PLS Toolbox®)
- Normalization order: 2, non-negativity tolerance: 10^{-5}, convergence tolerance: 10^{-8}, maximum number of iterations: 300.
- Initial guess method: Identify points on the exterior of a data space (exteriorpts – PLS Toolbox®), minimum norm: 0.03

4. PLS:

- Algorithm: SIMPLS,[4] confidence limit: 0.95.

5. SVM:

- Algorithm: libsvm,[5] kernel: rbf, svm type: epsilon-svr

6. Cross-validation

- Venetian blinds, data splits: 39 (training data size: 78), thickness: 1.

Supplementary Note

PLS and SVM. Simple regression methods such as partial least squares (PLS) and principal component regression (PCR) were previously utilized to build effective, sensitive and robust models for quantitative analysis in vibrational spectroscopy[6-9]. They effectively solve the linear regression problem

$$y = (\omega^t x_i) + b$$

by finding optimal ω and *b,* given training vectors x_i and observations *y* using the least squares approach:

$$min \sum_{i=1}^{m} \big(y_i \; - \; (\omega^t x_i + b)\big)^2$$

For their aptitude towards nonlinear or small sample-size problems, support vector machines (SVM) have recently found interest in classification or regression applications in spectroscopy and microscopy[10-14]. Initially introduced for pattern recognition, SVM aims to find an optimal hyperplane to separate different classes of data. SVM algorithms find optimal hyperplanes using data points closest to that hyperplane via support vectors[11, 15]. Support vector regression (SVR) was later introduced with the ε-insensitive loss function, allowing feasibility for the following convex optimization problem[16]:

$$min \frac{1}{2}\|\omega\|^2 + C\sum_{i=1}^{m}(\xi_i + \xi_i^*)$$

$$subject\ to \begin{cases} y_i - \big(\omega^t \phi(x_i)\big) - b \; \le \; \varepsilon + \xi_i \\ \big(\omega^t \phi(x_i)\big) + b - y_i \; \le \; \varepsilon + \xi_i^* \\ \xi_i, \xi_i^* \ge 0 \end{cases}$$

Here, the cost parameter, *C*, provides a control over the penalty associated with errors larger than ε and is optimized to allow accurate predictions without overfitting.

The above optimization problem can generally be solved more easily using a dual formulation by constructing a Lagrangian. For brevity, further mathematical formulation was not presented here, but can be found in the original work[16] and various applications of SVM.[9, 11, 17]

In the above equation, $\phi(x_i)$ is the kernel function, which is the inner product of ω^t and x_i in the simplest regard. Problems with high nonlinearity can be modeled with SVM, using kernel functions such as polynomial or radial basis functions. In radial basis function (rbf) kernel, the shape of separating hyperplanes can be controlled by gamma (γ) in the below equation:

$$K(x, y) = exp\{-\gamma\|x - y\|^2\}$$

In this work, we used ε-SVR with a radial basis function and optimized the parameters C and γ using a grid search.

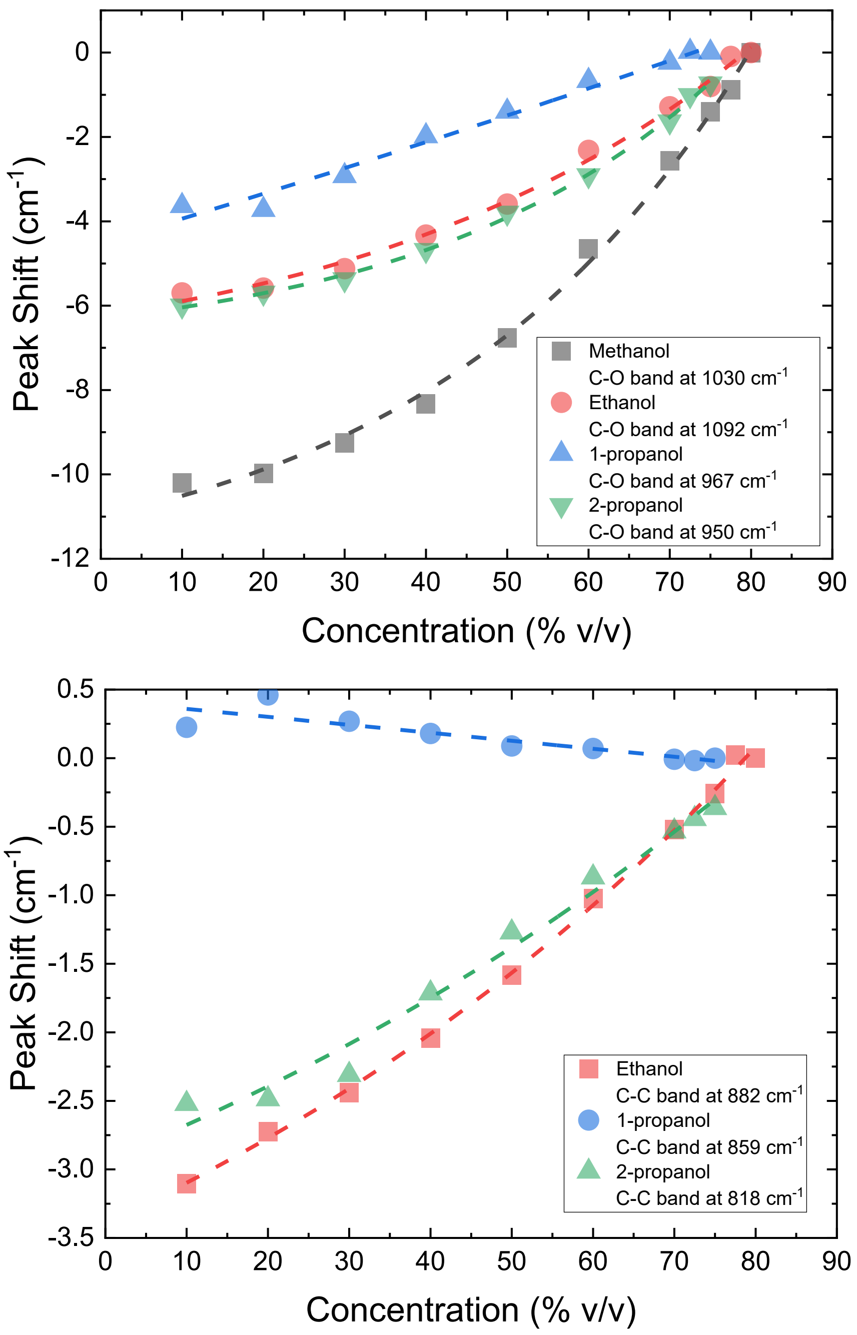

Supplementary Figure 1. Measured spectral shifts of C-O and C-C Raman bands of alcohols used for hand sanitizer formulations in this study when the water-alcohol amounts were varied.

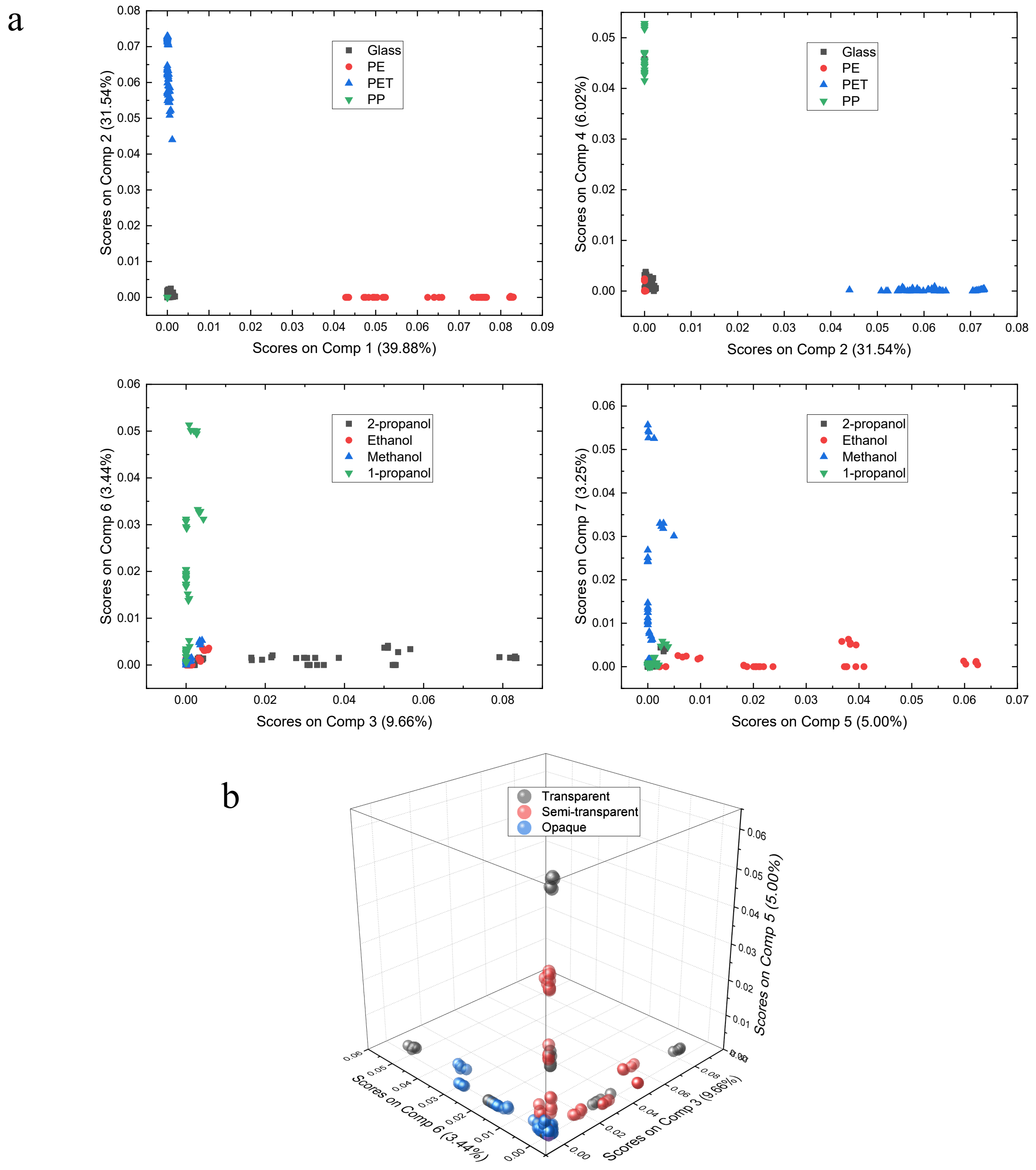

Supplementary Figure 2. MCR scores plots from traditional Raman spectra for various components. (**a**) 2D scores plots show that components 1,2, and 4 provide container based class separation whereas 3,5, 6, and 7 provide separation for alcohols, albeit this separation is limited for certain samples. (**b**) 3D scores, color-coded for container transparency and plotted on components representing the alcohols. Since axes represent contents, scores from transparent containers are found to be further from the origin compared with scores from semi-transparent and opaque containers.

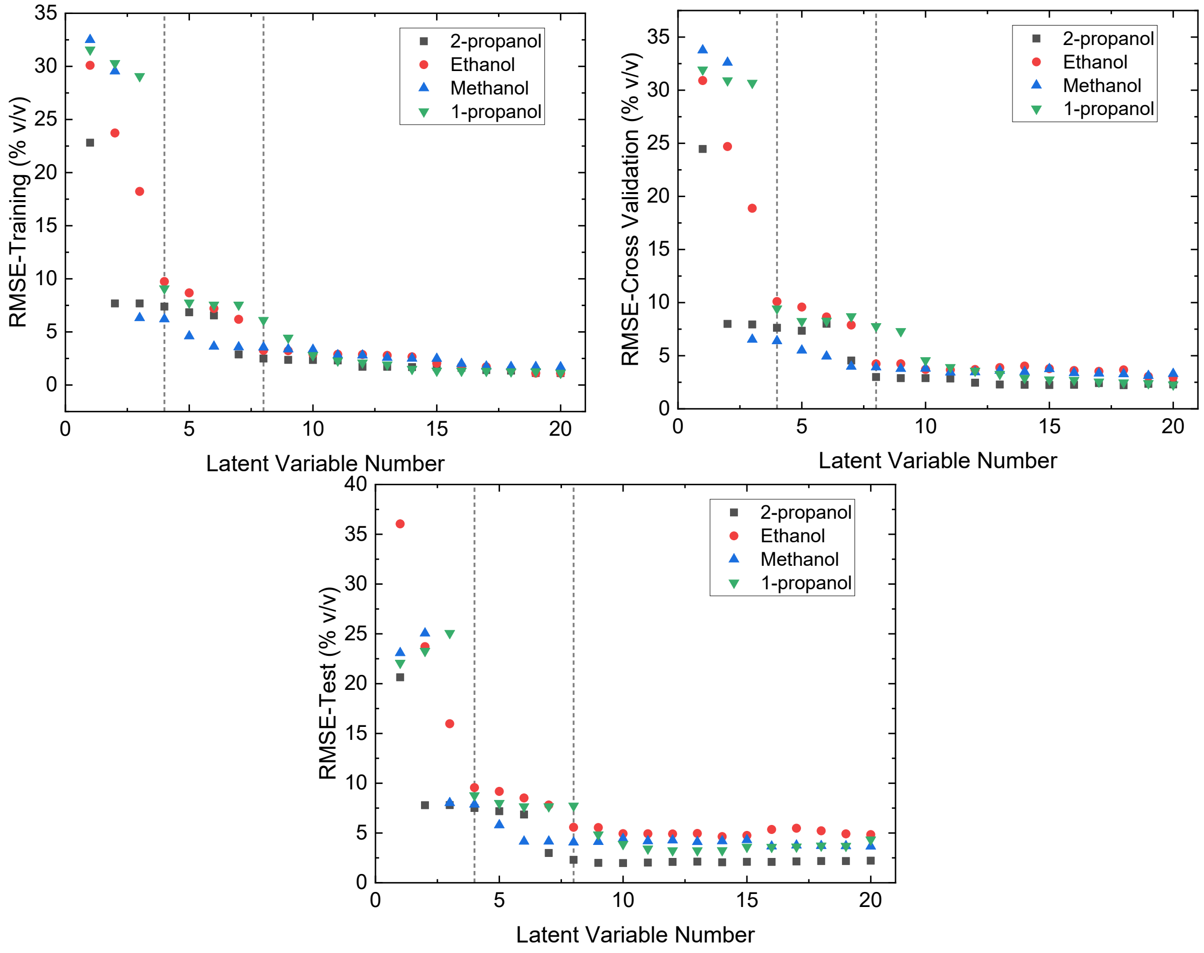

Supplementary Figure 3. RMSE for calibration, cross-validation and prediction as a function of latent variables in the PLS model. Based on the cross-validation RMSE, selection of 4 latent variables was found reasonable as shown with the dashed black line.

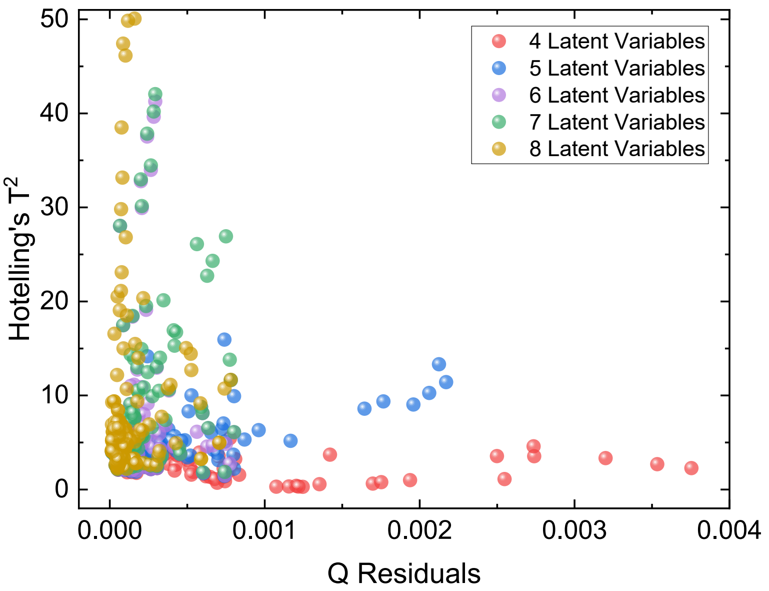

Supplementary Figure 4. Evolution of Q-residuals and T^2 statistics for the PLS model when the number of latent variables are changed from four to eight.

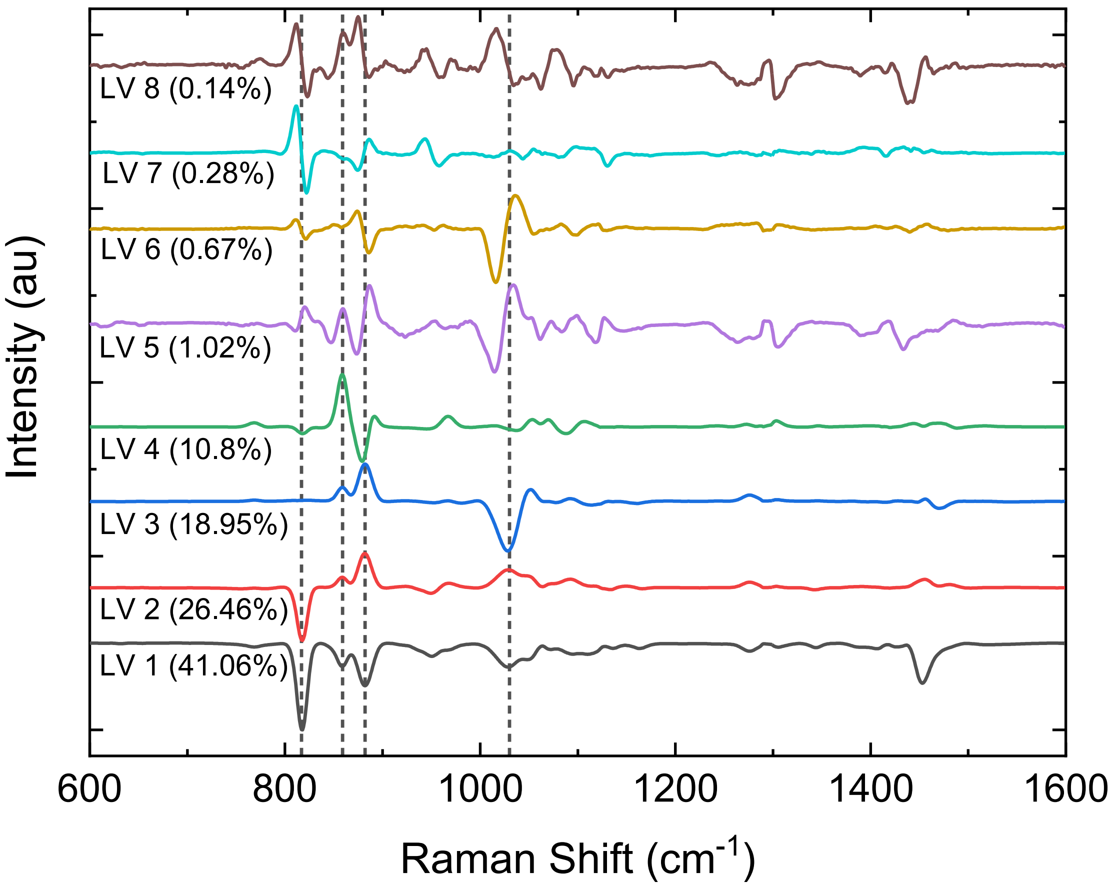

Supplementary Figure 5. First 7 latent variables in the PLS model. Vertical dashed lines indicate unique alcohol features: 2-propanol at 818 cm^{-1}, 1-propanol at 859 cm^{-1}, ethanol at 882 cm^{-1}, and methanol at 1030 cm^{-1}.

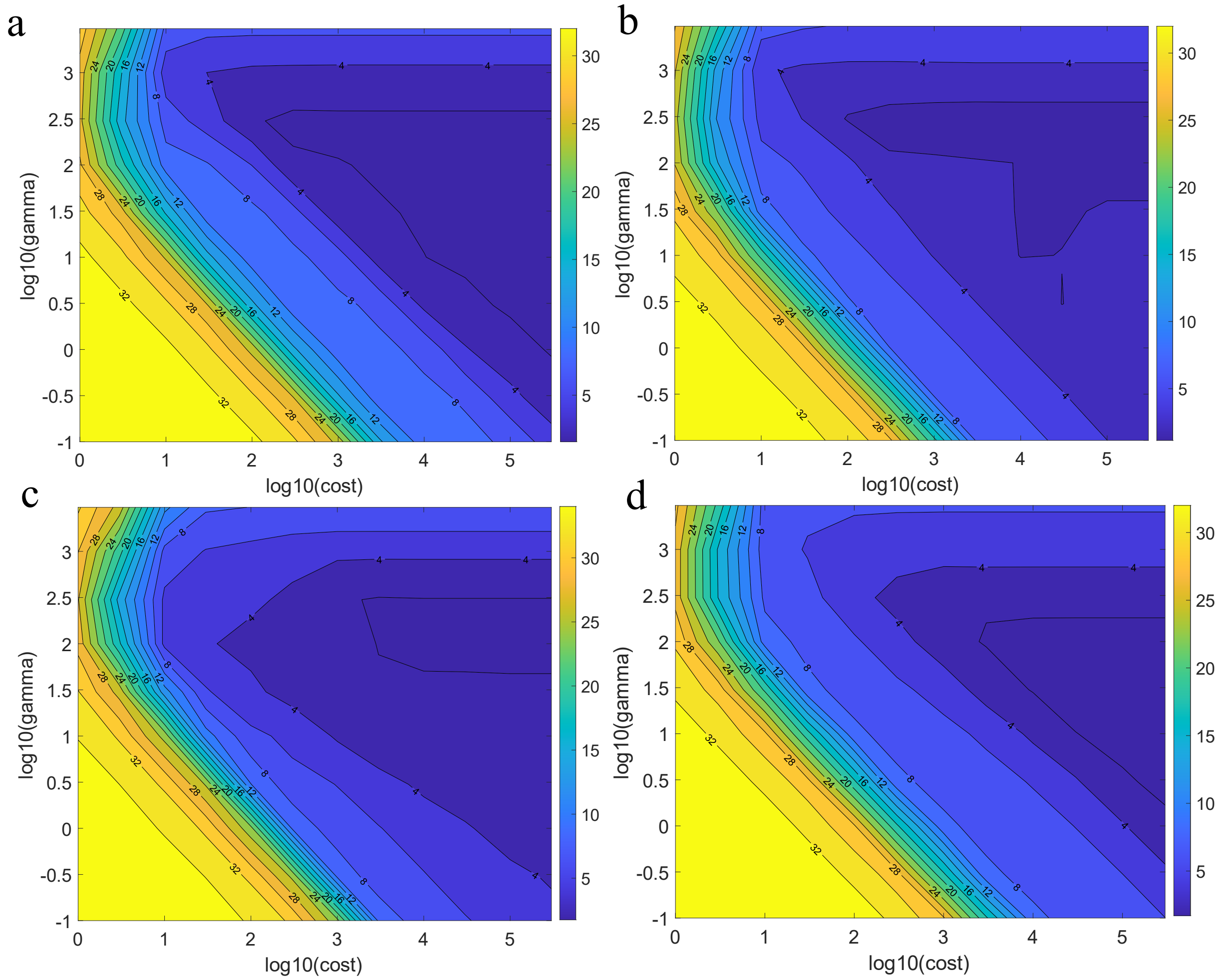

Supplementary Figure 6. Grid search plot for optimization of cost, C, and gamma, γ. For each alcohol a separate grid-search was performed. (**a**) 2-propanol, (**b**) Ethanol, (**c**) Methanol, (**d**) 1-propanol). In the final model, cost was chosen as 3×10^3 and gamma as 30.

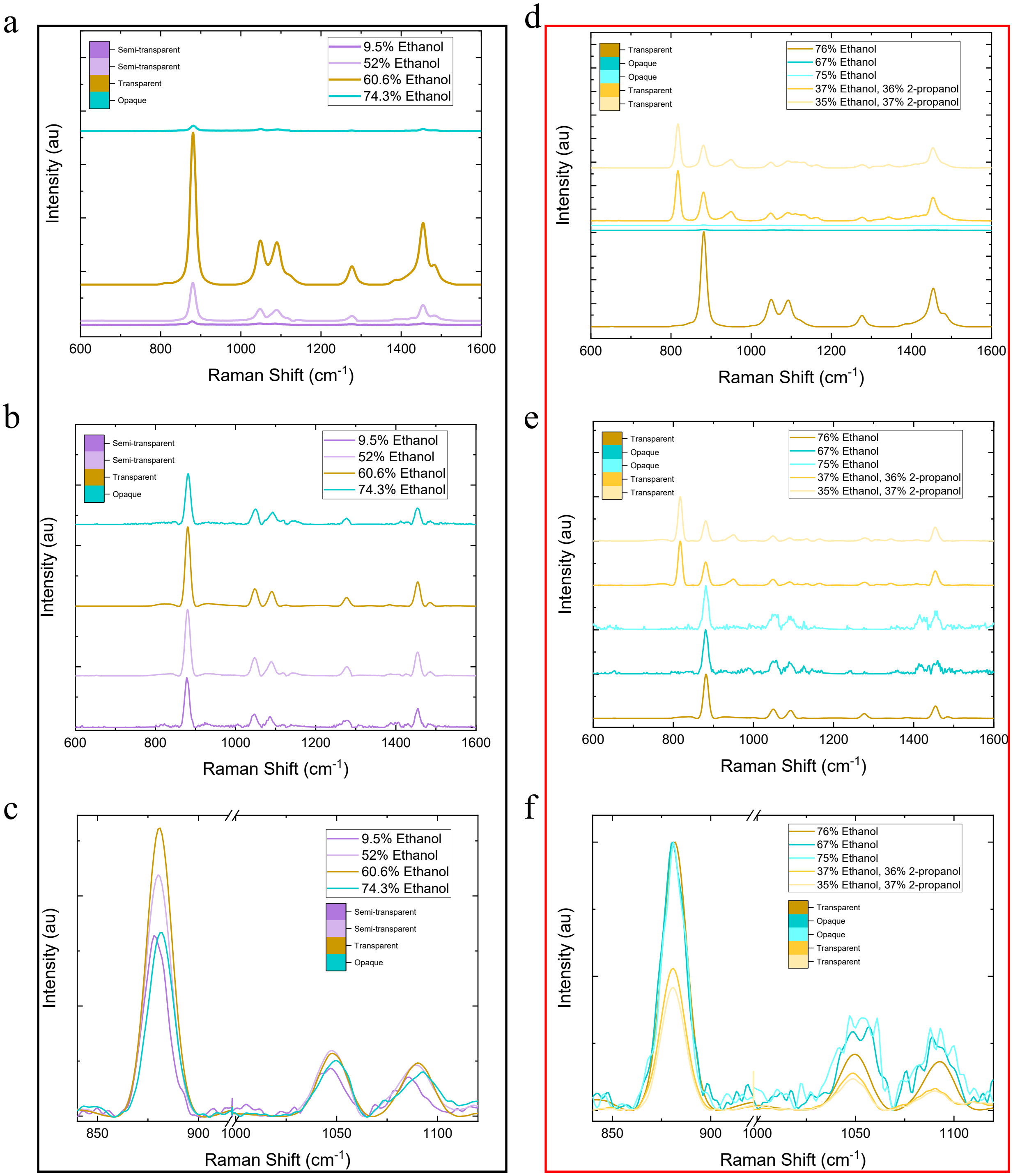

Supplementary Figure 7. Scaled-subtracted spectra of well (**a-c**) and poorly (**d-f**) predicted hand sanitizer products, before (**a, d**) and after (**b, e**) normalization. Spectra in top and middle panels are offset for clarity. Spectral shifts are resolved in (**c**) where prediction errors are minimal, whereas in (**f**) spectra is noisy and does not allow resolution of shifts.

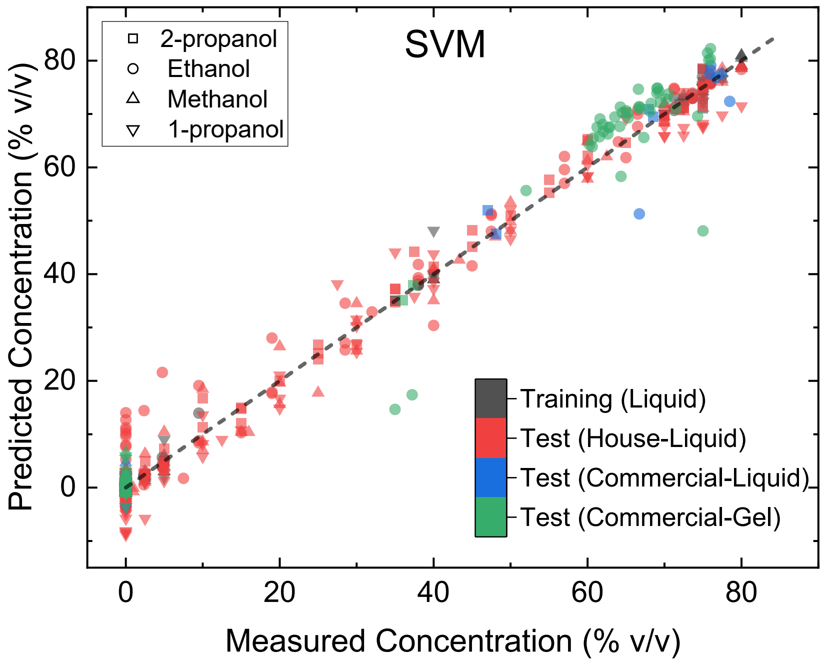

Supplementary Figure 8. Predicted alcohol concentrations plotted against measured values using the SVM model. Each symbol represents a separate alcohol, products are color coded based on the type of formulation (liquid, gel) and origin.

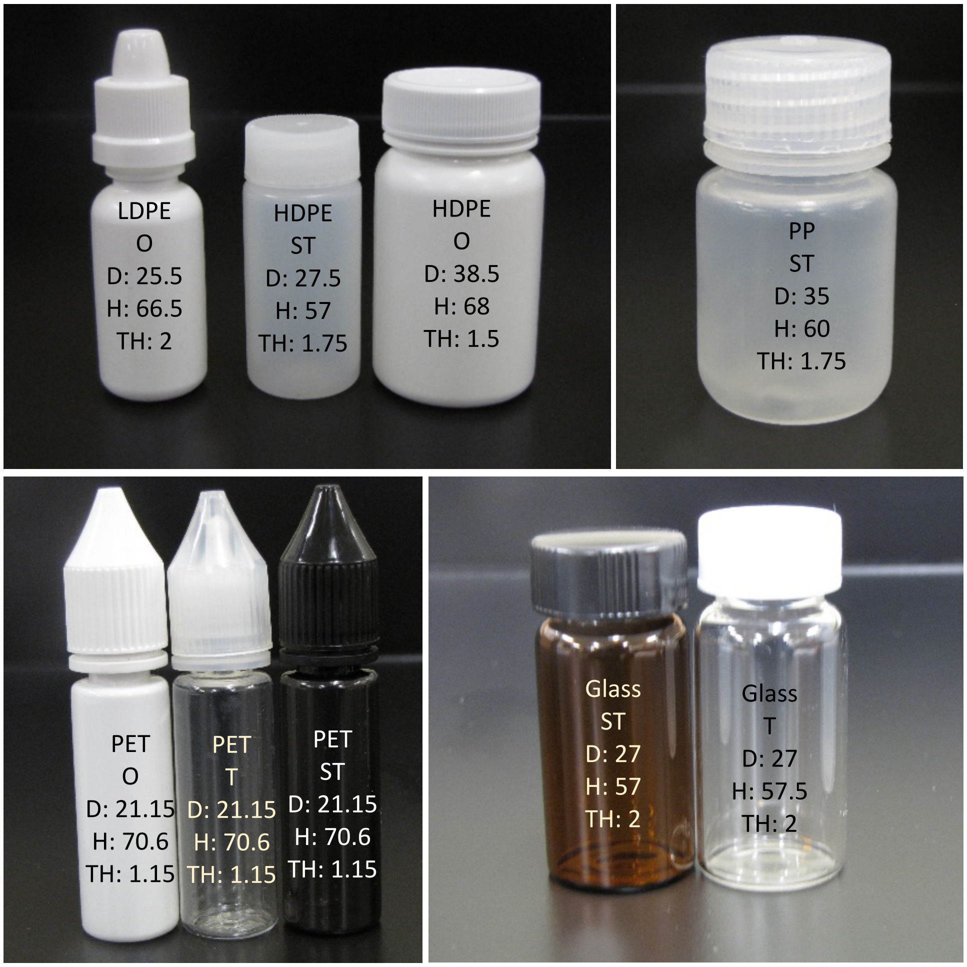

Supplementary Figure 9. Types of containers used for method development, testing, and storing of in-house hand sanitizers formulations. Dimensions are in mm, specifications are (O: Opaque, ST: Semi-transparent, T: Transparent, D: Diameter, H: Height, TH: Thickness).

a

b

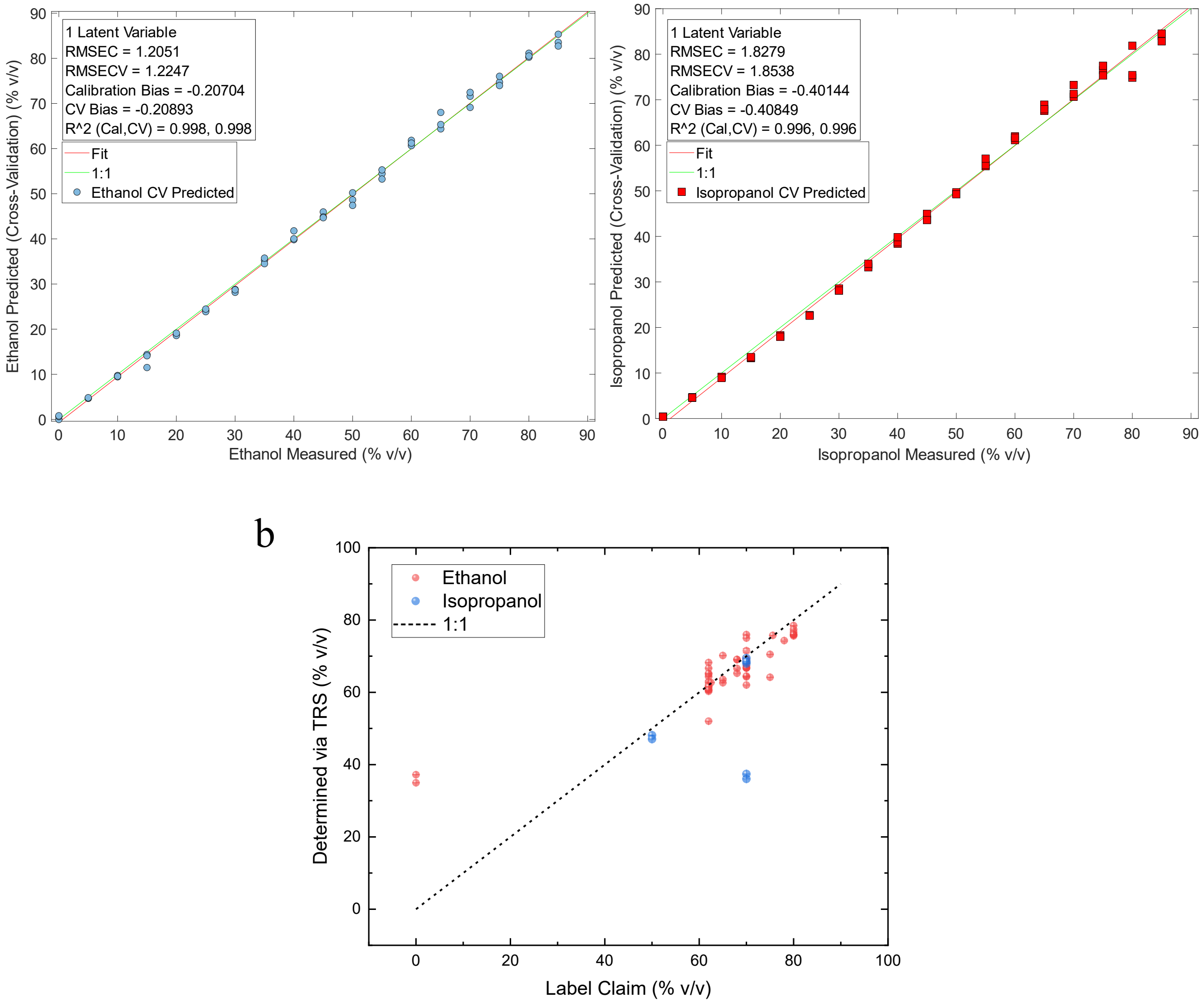

Supplementary Figure 10. (**a**) Linear regression results for the cross-validated training set used in the determination of alcohol content in commercial hand sanitizers using transmission Raman spectroscopy (TRS). (**b**) Comparison of label claim amounts and concentrations determined using the quantitative TRS method. Only 4 products were found to not match the label claim (no more than 10% above or 10% below).

Supplementary References

1. de Juan, A.; Jaumot, J.; Tauler, R., Multivariate Curve Resolution (MCR). Solving the mixture analysis problem. *Analytical Methods* **2014,** *6* (14), 4964-4976.

2. Xu, T.; Yilmaz, H.; Willett, D. R.; Strasinger, C.; Rodriguez, J. D.; Keire, D. A.; Wokovich, A. M., Raman mapping of fentanyl transdermal delivery systems with off-label modifications. *Analyst* **2019**.

3. Eilers, P. H. C., A Perfect Smoother. *Analytical Chemistry* **2003,** *75* (14), 3631-3636.

4. de Jong, S., SIMPLS: An alternative approach to partial least squares regression. *Chemometrics and Intelligent Laboratory Systems* **1993,** *18* (3), 251-263.

5. Chang, C.-C.; Lin, C.-J. LIBSVM: A Library for Support Vector Machines. https://www.csie.ntu.edu.tw/~cjlin/papers/libsvm.pdf.

6. Berger, A. J.; Itzkan, I.; Feld, M. S., Feasibility of measuring blood glucose concentration by near-infrared Raman spectroscopy. *Spectrochimica Acta Part A: Molecular and Biomolecular Spectroscopy* **1997,** *53* (2), 287-292.

7. Kachrimanis, K.; Braun, D. E.; Griesser, U. J., Quantitative analysis of paracetamol polymorphs in powder mixtures by FT-Raman spectroscopy and PLS regression. *J Pharm Biomed Anal* **2007,** *43* (2), 407-12.

8. Moros, J.; Garrigues, S.; de la Guardia, M., Evaluation of nutritional parameters in infant formulas and powdered milk by Raman spectroscopy. *Anal Chim Acta* **2007,** *593* (1), 30-8.

9. Thissen, U.; Pepers, M.; Üstün, B.; Melssen, W. J.; Buydens, L. M. C., Comparing support vector machines to PLS for spectral regression applications. *Chemometrics and Intelligent Laboratory Systems* **2004,** *73* (2), 169-179.

10. Baker, M. J.; Trevisan, J.; Bassan, P.; Bhargava, R.; Butler, H. J.; Dorling, K. M.; Fielden, P. R.; Fogarty, S. W.; Fullwood, N. J.; Heys, K. A.; Hughes, C.; Lasch, P.; Martin-Hirsch, P. L.; Obinaju, B.; Sockalingum, G. D.; Sule-Suso, J.; Strong, R. J.; Walsh, M. J.; Wood, B. R.; Gardner, P.; Martin, F. L., Using Fourier transform IR spectroscopy to analyze biological materials. *Nat Protoc* **2014,** *9* (8), 1771-91.

11. Li, Y.; Li, F.; Yang, X.; Guo, L.; Huang, F.; Chen, Z.; Chen, X.; Zheng, S., Quantitative analysis of glycated albumin in serum based on ATR-FTIR spectrum combined with SiPLS and SVM. *Spectrochim Acta A Mol Biomol Spectrosc* **2018,** *201*, 249-257.

12. Pyrgiotakis, G.; Kundakcioglu, O. E.; Finton, K.; Pardalos, P. M.; Powers, K.; Moudgil, B. M., Cell death discrimination with Raman spectroscopy and support vector machines. *Ann Biomed Eng* **2009,** *37* (7), 1464-73.

13. Sattlecker, M.; Bessant, C.; Smith, J.; Stone, N., Investigation of support vector machines and Raman spectroscopy for lymph node diagnostics. *Analyst* **2010,** *135* (5), 895-901.

14. Chen, B.; Lu, Y.; Pan, W.; Xiong, J.; Yang, Z.; Yan, W.; Liu, L.; Qu, J., Support Vector Machine Classification of Nonmelanoma Skin Lesions Based on Fluorescence Lifetime Imaging Microscopy. *Anal Chem* **2019,** *91* (16), 10640-10647.

15. Vapnik, V. N., *The nature of statistical learning theory*. Springer-Verlag: 1995.

16. Drucker, H.; Burges, C. J.; Kaufman, L.; Smola, A.; Vapnik, V., Support vector regression machines. *Advances in neural information processing systems* **1996,** *9*, 155-161.

17. Dong, W.; Zhang, Y.; Zhang, B.; Wang, X., Quantitative analysis of adulteration of extra virgin olive oil using Raman spectroscopy improved by Bayesian framework least squares support vector machines. *Analytical Methods* **2012,** *4* (9).
