# Supplementary material for: Through-container quantitative analysis of hand sanitizers using spatially offset Raman spectroscopy
Source: Commun Chem. 2021 Sep 2;4:126. doi: 10.1038/s42004-021-00563-6 (PMC9814617; doi:10.1038/s42004-021-00563-6)
Supplement: Supplementary file 3 — Supplementary Data 1 [file 42004_2021_563_MOESM3_ESM.docx]

**Supplementary Data 1.** Contents, formulations, and container types of samples used in MCR and regression models. ST: Semi-transparent, T: Transparent, O: Opaque.

|  | **Dataset** | **Alcohol content** | **Hand Sanitizer Formulation** | **Container Type** |
| --- | --- | --- | --- | --- |
| 1 | Training | 75% v/v 2-propanol | In-house, Liquid | HDPE-ST |
| 2 | Training | 75% v/v 2-propanol | In-house, Liquid | PET-O |
| 3 | Training | 75% v/v 2-propanol | In-house, Liquid | PET-T |
| 4 | Training | 75% v/v 2-propanol | In-house, Liquid | PET-ST |
| 5 | Training | 75% v/v 2-propanol | In-house, Liquid | LDPE-O |
| 6 | Training | 75% v/v 2-propanol | In-house, Liquid | HDPE-O |
| 7 | Training | 75% v/v 2-propanol | In-house, Liquid | Glass-T |
| 8 | Training | 75% v/v 2-propanol | In-house, Liquid | Glass- ST |
| 9 | Training | 75% v/v 2-propanol | In-house, Liquid | PP-ST |
| 10 | Training | 76% v/v Ethanol | In-house, Liquid | HDPE-ST |
| 11 | Training | 76% v/v Ethanol | In-house, Liquid | PET-O |
| 12 | Training | 76% v/v Ethanol | In-house, Liquid | PET-T |
| 13 | Training | 76% v/v Ethanol | In-house, Liquid | PET- ST |
| 14 | Training | 76% v/v Ethanol | In-house, Liquid | LDPE-O |
| 15 | Training | 76% v/v Ethanol | In-house, Liquid | HDPE- O |
| 16 | Training | 76% v/v Ethanol | In-house, Liquid | Glass- T |
| 17 | Training | 76% v/v Ethanol | In-house, Liquid | Glass-ST |
| 18 | Training | 76% v/v Ethanol | In-house, Liquid | PP-ST |
| 19 | Training | 80% v/v Methanol | In-house, Liquid | HDPE - ST |
| 20 | Training | 80% v/v Methanol | In-house, Liquid | PET- O |
| 21 | Training | 80% v/v Methanol | In-house, Liquid | PET- T |
| 22 | Training | 80% v/v Methanol | In-house, Liquid | PET- ST |
| 23 | Training | 80% v/v Methanol | In-house, Liquid | LDPE-O |
| 24 | Training | 80% v/v Methanol | In-house, Liquid | HDPE -O |
| 25 | Training | 80% v/v Methanol | In-house, Liquid | Glass- T |
| 26 | Training | 80% v/v Methanol | In-house, Liquid | Glass- ST |
| 27 | Training | 80% v/v Methanol | In-house, Liquid | PP-ST |
| 28 | Training | 75% v/v 1-propanol | In-house, Liquid | HDPE - ST |
| 29 | Training | 75% v/v 1-propanol | In-house, Liquid | PET - O |
| 30 | Training | 75% v/v 1-propanol | In-house, Liquid | PET- T |
| 31 | Training | 75% v/v 1-propanol | In-house, Liquid | PET - ST |
| 32 | Training | 75% v/v 1-propanol | In-house, Liquid | LDPE-O |
| 33 | Training | 75% v/v 1-propanol | In-house, Liquid | HDPE - O |
| 34 | Training | 75% v/v 1-propanol | In-house, Liquid | Glass - T |
| 35 | Training | 75% v/v 1-propanol | In-house, Liquid | Glass - ST |
| 36 | Training | 75% v/v 1-propanol | In-house, Liquid | PP-ST |
| 37 | Training | 0% alcohol | In-house, Liquid | HDPE-ST |
| 38 | Training | 2.375% v/v Ethanol | In-house, Liquid | HDPE-ST |
| 39 | Training | 4.75% v/v Ethanol | In-house, Liquid | HDPE-ST |
| 40 | Training | 9.5 % v/v Ethanol | In-house, Liquid | HDPE-ST |
| 41 | Training | 38% v/v Ethanol | In-house, Liquid | HDPE-ST |
| 42 | Training | 71.25% v/v Ethanol | In-house, Liquid | HDPE-ST |
| 43 | Training | 73.625% v/v Ethanol | In-house, Liquid | HDPE-ST |
| 44 | Training | 2.5% v/v Methanol | In-house, Liquid | HDPE-ST |
| 45 | Training | 5% v/v Methanol | In-house, Liquid | HDPE-ST |
| 46 | Training | 40% v/v Methanol | In-house, Liquid | HDPE-ST |
| 47 | Training | 75% v/v Methanol | In-house, Liquid | HDPE-ST |
| 48 | Training | 77.5% v/v Methanol | In-house, Liquid | HDPE-ST |
| 49 | Training | 2.5% 2-propanol | In-house, Liquid | HDPE-ST |
| 50 | Training | 5% v/v 2-propanol | In-house, Liquid | HDPE-ST |
| 51 | Training | 40% v/v 2-propanol | In-house, Liquid | HDPE-ST |
| 52 | Training | 70% v/v 2-propanol | In-house, Liquid | HDPE-ST |
| 53 | Training | 72.5% v/v 2-propanol | In-house, Liquid | HDPE-ST |
| 54 | Training | 2.5% v/v 1-propanol | In-house, Liquid | HDPE-ST |
| 55 | Training | 5% v/v 1-propanol | In-house, Liquid | HDPE-ST |
| 56 | Training | 40% v/v 1-propanol | In-house, Liquid | HDPE-ST |
| 57 | Training | 70% v/v 1-propanol | In-house, Liquid | HDPE-ST |
| 58 | Training | 72.5% v/v 1-propanol | In-house, Liquid | HDPE-ST |
| 59 | Training | 73.625 %v/v Ethanol + 2.5 %v/v Methanol | In-house, Liquid | HDPE-ST |
| 60 | Training | 71.25 %v/v Ethanol + 5 %v/v Methanol | In-house, Liquid | HDPE-ST |
| 61 | Training | 38 %v/v Ethanol + 40 %v/v Methanol | In-house, Liquid | HDPE-ST |
| 62 | Training | 4.75 %v/v Ethanol + 75 %v/v Methanol | In-house, Liquid | HDPE-ST |
| 63 | Training | 2.375 %v/v Ethanol + 77.5 %v/v Methanol | In-house, Liquid | HDPE-ST |
| 64 | Training | 73.625 %v/v Ethanol + 2.5 %v/v 1-propanol | In-house, Liquid | HDPE-ST |
| 65 | Training | 71.25 %v/v Ethanol + 5 %v/v 1-propanol | In-house, Liquid | HDPE-ST |
| 66 | Training | 38 %v/v Ethanol + 40 %v/v 1-propanol | In-house, Liquid | HDPE-ST |
| 67 | Training | 4.75 %v/v Ethanol + 75 %v/v 1-propanol | In-house, Liquid | HDPE-ST |
| 68 | Training | 2.375 %v/v Ethanol + 77.5 %v/v 1-propanol | In-house, Liquid | HDPE-ST |
| 69 | Training | 72.5 %v/v 2-propanol + 2.5 %v/v Methanol | In-house, Liquid | HDPE-ST |
| 70 | Training | 70 %v/v 2-propanol + 5 %v/v Methanol | In-house, Liquid | HDPE-ST |
| 71 | Training | 35 %v/v 2-propanol + 40 %v/v Methanol | In-house, Liquid | HDPE-ST |
| 72 | Training | 5 %v/v 2-propanol + 70 %v/v Methanol | In-house, Liquid | HDPE-ST |
| 73 | Training | 2.5 %v/v 2-propanol + 72.5 %v/v Methanol | In-house, Liquid | HDPE-ST |
| 74 | Training | 72.5 %v/v 2-propanol + 2.5 %v/v 1-propanol | In-house, Liquid | HDPE-ST |
| 75 | Training | 70 %v/v 2-propanol + 5 %v/v 1-propanol | In-house, Liquid | HDPE-ST |
| 76 | Training | 35 %v/v 2-propanol + 40 %v/v 1-propanol | In-house, Liquid | HDPE-ST |
| 77 | Training | 5 %v/v 2-propanol + 70 %v/v 1-propanol | In-house, Liquid | HDPE-ST |
| 78 | Training | 2.5 %v/v 2-propanol + 72.5 %v/v 1-propanol | In-house, Liquid | HDPE-ST |
| 79 | Test | 25% v/v 2-propanol + 40% v/v Ethanol + 12.5 v/v 1-propanol | In-house, Liquid | HDPE-O |
| 80 | Test | 25% v/v Methanol | In-house, Liquid | HDPE-O |
| 81 | Test | 10% 2-propanol + 7.5 % Ethanol + 27.5 % 1-propanol | In-house, Liquid | PP-ST |
| 82 | Test | 62.5 %v/v Methanol | In-house, Liquid | PP-ST |
| 83 | Test | 32 % v/v Ethanol + 48% v/v Methanol | In-house, Liquid | LDPE-O |
| 84 | Test | 15% 2-propanol | In-house, Liquid | LDPE-O |
| 85 | Test | 60 % 2-propanol + 15% v/v 1-propanol | In-house, Liquid | PET-O |
| 86 | Test | 65 % v/v Ethanol | In-house, Liquid | PET-ST |
| 87 | Test | 37.5 % v/v 2-propanol + 37.5 %v/v 1-propanol | In-house, Liquid | PET-ST |
| 88 | Test | 64% v/v Ethanol + 16% v/v Methanol | In-house, Liquid | PET-T |
| 89 | Test | 60% v/v 2-propanol + 15% v/v Methanol | In-house, Liquid | PET-T |
| 90 | Test | 45 % Ethanol + 35% 1-propanol | In-house, Liquid | Glass-T |
| 91 | Test | 15 % Ethanol + 65% 1-propanol | In-house, Liquid | Glass-T |
| 92 | Test | 43.34 % v/v Methanol | In-house, Liquid | Glass-ST |
| 93 | Test | 80% v/v Ethanol + 1% v/v Methanol | In-house, Liquid | Glass-ST |
| 94 | Test | 75% v/v 2-propanol +1% v/v Methanol | In-house, Liquid | HDPE-ST |
| 95 | Test | 30% v/v 1-propanol | In-house, Liquid | HDPE-ST |
| 96 | Test | 35% 2-propanol | In-house, Liquid | HDPE-ST |
| 97 | Test | 0% alcohol | In-house, Liquid | HDPE-ST |
| 98 | Test | 2.375% v/v Ethanol | In-house, Liquid | HDPE-ST |
| 99 | Test | 4.75% v/v Ethanol | In-house, Liquid | HDPE-ST |
| 100 | Test | 9.5% v/v Ethanol | In-house, Liquid | HDPE-ST |
| 101 | Test | 19% v/v Ethanol | In-house, Liquid | HDPE-ST |
| 102 | Test | 28.5% v/v Ethanol | In-house, Liquid | HDPE-ST |
| 103 | Test | 38% v/v Ethanol | In-house, Liquid | HDPE-ST |
| 104 | Test | 47.5% v/v Ethanol | In-house, Liquid | HDPE-ST |
| 105 | Test | 57% v/v Ethanol | In-house, Liquid | HDPE-ST |
| 106 | Test | 66.5% v/v Ethanol | In-house, Liquid | HDPE-ST |
| 107 | Test | 71.25% v/v Ethanol | In-house, Liquid | HDPE-ST |
| 108 | Test | 73.625% v/v Ethanol | In-house, Liquid | HDPE-ST |
| 109 | Test | 76% v/v Ethanol | In-house, Liquid | HDPE-ST |
| 110 | Test | 0% alcohol | In-house, Liquid | HDPE-ST |
| 111 | Test | 2.5 % 2-propanol | In-house, Liquid | HDPE-ST |
| 112 | Test | 5 % 2-propanol | In-house, Liquid | HDPE-ST |
| 113 | Test | 10 % 2-propanol | In-house, Liquid | HDPE-ST |
| 114 | Test | 20 % 2-propanol | In-house, Liquid | HDPE-ST |
| 115 | Test | 30 % 2-propanol | In-house, Liquid | HDPE-ST |
| 116 | Test | 40 % 2-propanol | In-house, Liquid | HDPE-ST |
| 117 | Test | 50 % 2-propanol | In-house, Liquid | HDPE-ST |
| 118 | Test | 60 % 2-propanol | In-house, Liquid | HDPE-ST |
| 119 | Test | 70 % 2-propanol | In-house, Liquid | HDPE-ST |
| 120 | Test | 72.5 % 2-propanol | In-house, Liquid | HDPE-ST |
| 121 | Test | 75 % 2-propanol | In-house, Liquid | HDPE-ST |
| 122 | Test | 0% alcohol | In-house, Liquid | HDPE-ST |
| 123 | Test | 2.5 % v/v Methanol | In-house, Liquid | HDPE-ST |
| 124 | Test | 5 % v/v Methanol | In-house, Liquid | HDPE-ST |
| 125 | Test | 10 % v/v Methanol | In-house, Liquid | HDPE-ST |
| 126 | Test | 20 % v/v Methanol | In-house, Liquid | HDPE-ST |
| 127 | Test | 30 % v/v Methanol | In-house, Liquid | HDPE-ST |
| 128 | Test | 50 % v/v Methanol | In-house, Liquid | HDPE-ST |
| 129 | Test | 60 % v/v Methanol | In-house, Liquid | HDPE-ST |
| 130 | Test | 70 % v/v Methanol | In-house, Liquid | HDPE-ST |
| 131 | Test | 75 % v/v Methanol | In-house, Liquid | HDPE-ST |
| 132 | Test | 77.5 % v/v Methanol | In-house, Liquid | HDPE-ST |
| 133 | Test | 80 % v/v Methanol | In-house, Liquid | HDPE-ST |
| 134 | Test | 40 % v/v Methanol | In-house, Liquid | HDPE-ST |
| 135 | Test | 0% alcohol | In-house, Liquid | HDPE-ST |
| 136 | Test | 2.5 % v/v 1-propanol | In-house, Liquid | HDPE-ST |
| 137 | Test | 5 % v/v 1-propanol | In-house, Liquid | HDPE-ST |
| 138 | Test | 10 % v/v 1-propanol | In-house, Liquid | HDPE-ST |
| 139 | Test | 20 % v/v 1-propanol | In-house, Liquid | HDPE-ST |
| 140 | Test | 30 % v/v 1-propanol | In-house, Liquid | HDPE-ST |
| 141 | Test | 40 % v/v 1-propanol | In-house, Liquid | HDPE-ST |
| 142 | Test | 50 % v/v 1-propanol | In-house, Liquid | HDPE-ST |
| 143 | Test | 60 % v/v 1-propanol | In-house, Liquid | HDPE-ST |
| 144 | Test | 70 % v/v 1-propanol | In-house, Liquid | HDPE-ST |
| 145 | Test | 72.5 % v/v 1-propanol | In-house, Liquid | HDPE-ST |
| 146 | Test | 75 % v/v 1-propanol | In-house, Liquid | HDPE-ST |
| 147 | Test | 76% v/v Ethanol | In-house, Liquid | HDPE-ST |
| 148 | Test | 76% v/v Ethanol | In-house, Liquid | HDPE-ST |
| 149 | Test | 73.625 %v/v Ethanol + 2.5 %v/v Methanol | In-house, Liquid | HDPE-ST |
| 150 | Test | 71.25 %v/v Ethanol + 5 %v/v Methanol | In-house, Liquid | HDPE-ST |
| 151 | Test | 66.5 %v/v Ethanol + 10 %v/v Methanol | In-house, Liquid | HDPE-ST |
| 152 | Test | 57 %v/v Ethanol + 20 %v/v Methanol | In-house, Liquid | HDPE-ST |
| 153 | Test | 47.5 %v/v Ethanol + 30 %v/v Methanol | In-house, Liquid | HDPE-ST |
| 154 | Test | 38 %v/v Ethanol + 40 %v/v Methanol | In-house, Liquid | HDPE-ST |
| 155 | Test | 28.5 %v/v Ethanol + 50 %v/v Methanol | In-house, Liquid | HDPE-ST |
| 156 | Test | 19 %v/v Ethanol + 60 %v/v Methanol | In-house, Liquid | HDPE-ST |
| 157 | Test | 9.5 %v/v Ethanol + 70 %v/v Methanol | In-house, Liquid | HDPE-ST |
| 158 | Test | 4.75 %v/v Ethanol + 75 %v/v Methanol | In-house, Liquid | HDPE-ST |
| 159 | Test | 2.375 %v/v Ethanol + 77.5 %v/v Methanol | In-house, Liquid | HDPE-ST |
| 160 | Test | 80% v/v Methanol | In-house, Liquid | HDPE-ST |
| 161 | Test | 75% v/v 2-propanol | In-house, Liquid | HDPE-ST |
| 162 | Test | 72.5 % v/v 2-propanol + 2.5 % Methanol | In-house, Liquid | HDPE-ST |
| 163 | Test | 70 % v/v 2-propanol + 5 % Methanol | In-house, Liquid | HDPE-ST |
| 164 | Test | 65 % v/v 2-propanol + 10 % Methanol | In-house, Liquid | HDPE-ST |
| 165 | Test | 55 % v/v 2-propanol + 20 % Methanol | In-house, Liquid | HDPE-ST |
| 166 | Test | 45 % v/v 2-propanol + 30 % Methanol | In-house, Liquid | HDPE-ST |
| 167 | Test | 35 % v/v 2-propanol + 40 % Methanol | In-house, Liquid | HDPE-ST |
| 168 | Test | 25 % v/v 2-propanol + 50 % Methanol | In-house, Liquid | HDPE-ST |
| 169 | Test | 15 % v/v 2-propanol + 60 % Methanol | In-house, Liquid | HDPE-ST |
| 170 | Test | 5% v/v 2-propanol + 70% Methanol | In-house, Liquid | HDPE-ST |
| 171 | Test | 2.5 % v/v 2-propanol + 72.5 % Methanol | In-house, Liquid | HDPE-ST |
| 172 | Test | 75% v/v Methanol | In-house, Liquid | HDPE-ST |
| 173 | Test | 76 % v/v Ethanol | In-house, Liquid | HDPE-ST |
| 174 | Test | 73.625 %v/v Ethanol + 2.5 %v/v 1-propanol | In-house, Liquid | HDPE-ST |
| 175 | Test | 71.25 %v/v Ethanol + 5 %v/v 1-propanol | In-house, Liquid | HDPE-ST |
| 176 | Test | 66.5 %v/v Ethanol + 10 % v/v 1-propanol | In-house, Liquid | HDPE-ST |
| 177 | Test | 57 %v/v Ethanol + 20 %v/v 1-propanol | In-house, Liquid | HDPE-ST |
| 178 | Test | 47.5 %v/v Ethanol + 30 %v/v 1-propanol | In-house, Liquid | HDPE-ST |
| 179 | Test | 38 %v/v Ethanol + 40 %v/v 1-propanol | In-house, Liquid | HDPE-ST |
| 180 | Test | 28.5 %v/v Ethanol + 50 %v/v 1-propanol | In-house, Liquid | HDPE-ST |
| 181 | Test | 19 %v/v Ethanol + 60 %v/v 1-propanol | In-house, Liquid | HDPE-ST |
| 182 | Test | 9.5 %v/v Ethanol + 70 %v/v 1-propanol | In-house, Liquid | HDPE-ST |
| 183 | Test | 4.75 %v/v Ethanol + 75 %v/v 1-propanol | In-house, Liquid | HDPE-ST |
| 184 | Test | 2.375 %v/v Ethanol + 77.5 %v/v 1-propanol | In-house, Liquid | HDPE-ST |
| 185 | Test | 80% v/v 1-propanol | In-house, Liquid | HDPE-ST |
| 186 | Test | 75 % v/v 2-propanol | In-house, Liquid | HDPE-ST |
| 187 | Test | 75 % v/v 2-propanol | In-house, Liquid | HDPE-ST |
| 188 | Test | 72.5 % v/v 2-propanol + 2.5 %v/v 1-propanol | In-house, Liquid | HDPE-ST |
| 189 | Test | 70 % v/v 2-propanol + 5 %v/v 1-propanol | In-house, Liquid | HDPE-ST |
| 190 | Test | 65 % v/v 2-propanol + 10 %v/v 1-propanol | In-house, Liquid | HDPE-ST |
| 191 | Test | 55 % v/v 2-propanol + 20 %v/v 1-propanol | In-house, Liquid | HDPE-ST |
| 192 | Test | 45 % v/v 2-propanol + 30 %v/v 1-propanol | In-house, Liquid | HDPE-ST |
| 193 | Test | 35 % v/v 2-propanol + 40 %v/v 1-propanol | In-house, Liquid | HDPE-ST |
| 194 | Test | 25 % v/v 2-propanol + 50 %v/v 1-propanol | In-house, Liquid | HDPE-ST |
| 195 | Test | 15 % v/v 2-propanol + 60 %v/v 1-propanol | In-house, Liquid | HDPE-ST |
| 196 | Test | 5 % v/v 2-propanol + 70 %v/v 1-propanol | In-house, Liquid | HDPE-ST |
| 197 | Test | 2.5 % v/v 2-propanol + 72.5 %v/v 1-propanol | In-house, Liquid | HDPE-ST |
| 198 | Test | 75 %v/v 1-propanol | In-house, Liquid | HDPE-ST |
| 199 | Test | 75.95 %v/v Ethanol | Commercial, Gel | HDPE-ST |
| 200 | Test | 75.76 %v/v Ethanol | Commercial, Gel | PETE-T |
| 201 | Test | 62.64 %v/v Ethanol | Commercial, Gel | PET-T |
| 202 | Test | 75.60 %v/v Ethanol | Commercial, Gel | PET-T |
| 203 | Test | 62.90 %v/v Ethanol | Commercial, Gel | PET-T |
| 204 | Test | 75.98 %v/v Ethanol | Commercial, Gel | PET-T |
| 205 | Test | 60.28 %v/v Ethanol | Commercial, Gel | PETE-T |
| 206 | Test | 64.27 %v/v Ethanol | Commercial, Gel | PET-T |
| 207 | Test | 64.52 %v/v Ethanol | Commercial, Gel | PET-T |
| 208 | Test | 65.02 %v/v Ethanol | Commercial, Gel | PET-T |
| 209 | Test | 63.56 %v/v Ethanol | Commercial, Gel | PET-T |
| 210 | Test | 69.48 %v/v 2-propanol | Commercial, Liquid | PET-T |
| 211 | Test | 61.56 %v/v Ethanol | Commercial, Gel | PET-ST |
| 212 | Test | 61.24 %v/v Ethanol | Commercial, Gel | PET-ST |
| 213 | Test | 60.74 %v/v Ethanol | Commercial, Gel | PET-ST |
| 214 | Test | 61.97 %v/v Ethanol | Commercial, Gel | PET-ST |
| 215 | Test | 61.63 %v/v Ethanol | Commercial, Gel | PET-ST |
| 216 | Test | 69.27 %v/v Ethanol | Commercial, Gel | PET-T |
| 217 | Test | 69.01 %v/v Ethanol | Commercial, Gel | PET-T |
| 218 | Test | 64.34 %v/v Ethanol | Commercial, Gel | PETE-O |
| 219 | Test | 68.26 %v/v Ethanol | Commercial, Gel | PET-T |
| 220 | Test | 66.67 %v/v Ethanol | Commercial, Gel | PET-T |
| 221 | Test | 70.51 %v/v Ethanol | Commercial, Gel | PET-T |
| 222 | Test | 67.99 %v/v 2-propanol | Commercial, Liquid | PET-T |
| 223 | Test | 65.27 %v/v Ethanol | Commercial, Gel | PET-T |
| 224 | Test | 66.61 %v/v Ethanol | Commercial, Gel | PET-T |
| 225 | Test | 69.03 %v/v Ethanol | Commercial, Gel | PET-T |
| 226 | Test | 69.12 %v/v Ethanol | Commercial, Gel | PET-T |
| 227 | Test | 71.52 %v/v Ethanol | Commercial, Gel | PETE-T |
| 228 | Test | 60.61 %v/v Ethanol | Commercial, Gel | PETE-T |
| 229 | Test | 70.20 %v/v Ethanol | Commercial, Gel | PET-T |
| 230 | Test | 67.33 %v/v Ethanol | Commercial, Gel | Plastic Tube-O |
| 231 | Test | 74.31 %v/v Ethanol | Commercial, Gel | Plastic Tube-O |
| 232 | Test | 78.48 %v/v Ethanol | Commercial, Liquid | PET-White |
| 233 | Test | 66.76 %v/v Ethanol | Commercial, Gel | PETE-T |
| 234 | Test | 66.73 %v/v Ethanol | Commercial, Liquid | HDPE-O |
| 235 | Test | 75.01 %v/v Ethanol | Commercial, Gel | HDPE-O |
| 236 | Test | 65.24 %v/v Ethanol | Commercial, Gel | HDPE-ST |
| 237 | Test | 48.14 %v/v 2-propanol | Commercial, Liquid | HDPE-ST |
| 238 | Test | 47.057 %v/v 2-propanol | Commercial, Liquid | HDPE-ST |
| 239 | Test | 68.53 %v/v 2-propanol | Commercial, Liquid | HDPE-ST |
| 240 | Test | 75.87 %v/v Ethanol | Commercial, Liquid | HDPE-ST |
| 241 | Test | 68.14 %v/v Ethanol | Commercial, Gel | HDPE-ST |
| 242 | Test | 76.65 %v/v Ethanol | Commercial, Liquid | HDPE-ST |
| 243 | Test | 62.71 %v/v Ethanol | Commercial, Gel | HDPE-ST |
| 244 | Test | 52.02 %v/v Ethanol | Commercial, Gel | HDPE-ST |
| 245 | Test | 77.40 %v/v Ethanol | Commercial, Liquid | HDPE-ST |
| 246 | Test | 76.02 %v/v Ethanol | Commercial, Liquid | PET-T |
| 247 | Test | 66.78 %v/v Ethanol | Commercial, Gel | HDPE-ST |
| 248 | Test | 64.17 %v/v Ethanol | Commercial, Gel | Plastic Tube-O |
| 249 | Test | 37.21 %v/v Ethanol + 36.00 %v/v 2-propanol | Commercial, Gel | Plastic Pouch-T |
| 250 | Test | 34.98 %v/v Ethanol + 37.38 %v/v 2-propanol | Commercial, Gel | Plastic Pouch-T |
| 251 | Test | 62.03 %v/v Ethanol | Commercial, Gel | HDPE-ST |
